# Supplementary material for: Modeling glioblastoma heterogeneity as a dynamic network of cell states
Source: Mol Syst Biol. 2021 Sep 16;17(9):e10105. doi: 10.15252/msb.202010105 (PMC8444284; doi:10.15252/msb.202010105)
Supplement: Supplementary file 6 — Source Data for Figure 5 [file MSB-17-e10105-s004.zip › Figure5A_sourcedata/GSEA_3071/nosigdb_stateV.GseaPreranked.1622795159954/DOROSZKO_U3180MG_CLUSTER_3.html]

Details for gene set DOROSZKO\_U3180MG\_CLUSTER\_3[GSEA]

|  || Dataset | state43071 |
| Phenotype | NoPhenotypeAvailable |
| Upregulated in class | na\_pos |
| GeneSet | DOROSZKO\_U3180MG\_CLUSTER\_3 |
| Enrichment Score (ES) | 0.699453 |
| Normalized Enrichment Score (NES) | 4.649741 |
| Nominal p-value | 0.0 |
| FDR q-value | 0.0 |
| FWER p-Value | 0.0 |
Table: GSEA Results Summary

  

Fig 1: Enrichment plot: DOROSZKO\_U3180MG\_CLUSTER\_3      
 Profile of the Running ES Score & Positions of GeneSet Members on the Rank Ordered List

  

| PROBE | GENE SYMBOL | GENE\_TITLE | RANK IN GENE LIST | RANK METRIC SCORE | RUNNING ES | CORE ENRICHMENT || 1 | HIST1H4C |  |  | 1 | 2.442 | 0.0067 | Yes |
| 2 | ATAD2 |  |  | 2 | 2.214 | 0.0130 | Yes |
| 3 | ESCO2 |  |  | 3 | 2.193 | 0.0192 | Yes |
| 4 | FAM111B |  |  | 4 | 2.012 | 0.0249 | Yes |
| 5 | HMGB2 |  |  | 5 | 2.011 | 0.0306 | Yes |
| 6 | NUSAP1 |  |  | 6 | 2.001 | 0.0363 | Yes |
| 7 | CCP110 |  |  | 9 | 1.920 | 0.0413 | Yes |
| 8 | PCNA |  |  | 10 | 1.908 | 0.0467 | Yes |
| 9 | TOP2A |  |  | 14 | 1.842 | 0.0513 | Yes |
| 10 | HIST1H1E |  |  | 15 | 1.830 | 0.0565 | Yes |
| 11 | ASRGL1 |  |  | 16 | 1.819 | 0.0617 | Yes |
| 12 | MCM4 |  |  | 17 | 1.815 | 0.0668 | Yes |
| 13 | DHFR |  |  | 18 | 1.795 | 0.0719 | Yes |
| 14 | HIST1H1D |  |  | 19 | 1.786 | 0.0770 | Yes |
| 15 | C21orf58 |  |  | 20 | 1.772 | 0.0820 | Yes |
| 16 | NCAPG |  |  | 21 | 1.760 | 0.0870 | Yes |
| 17 | RAD51AP1 |  |  | 22 | 1.757 | 0.0920 | Yes |
| 18 | FAM111A |  |  | 24 | 1.738 | 0.0967 | Yes |
| 19 | H2AFX |  |  | 26 | 1.725 | 0.1014 | Yes |
| 20 | PCLAF |  |  | 27 | 1.720 | 0.1063 | Yes |
| 21 | CENPF |  |  | 28 | 1.705 | 0.1111 | Yes |
| 22 | TYMS |  |  | 30 | 1.683 | 0.1157 | Yes |
| 23 | CCNE2 |  |  | 32 | 1.664 | 0.1202 | Yes |
| 24 | RRM2 |  |  | 34 | 1.660 | 0.1247 | Yes |
| 25 | MYBL2 |  |  | 35 | 1.651 | 0.1294 | Yes |
| 26 | CENPU |  |  | 36 | 1.645 | 0.1340 | Yes |
| 27 | KNL1 |  |  | 38 | 1.629 | 0.1384 | Yes |
| 28 | SGO1 |  |  | 40 | 1.614 | 0.1428 | Yes |
| 29 | E2F1 |  |  | 44 | 1.602 | 0.1467 | Yes |
| 30 | MKI67 |  |  | 45 | 1.598 | 0.1513 | Yes |
| 31 | BRCA1 |  |  | 46 | 1.592 | 0.1558 | Yes |
| 32 | GNG4 |  |  | 47 | 1.590 | 0.1603 | Yes |
| 33 | HELLS |  |  | 48 | 1.588 | 0.1648 | Yes |
| 34 | MCM3 |  |  | 49 | 1.586 | 0.1693 | Yes |
| 35 | XRCC2 |  |  | 50 | 1.574 | 0.1737 | Yes |
| 36 | KIF15 |  |  | 52 | 1.568 | 0.1780 | Yes |
| 37 | FOXM1 |  |  | 53 | 1.550 | 0.1824 | Yes |
| 38 | PBK |  |  | 54 | 1.536 | 0.1867 | Yes |
| 39 | EZH2 |  |  | 55 | 1.534 | 0.1911 | Yes |
| 40 | GINS2 |  |  | 57 | 1.528 | 0.1952 | Yes |
| 41 | EMP2 |  |  | 59 | 1.511 | 0.1993 | Yes |
| 42 | DTL |  |  | 60 | 1.503 | 0.2035 | Yes |
| 43 | MXD3 |  |  | 62 | 1.500 | 0.2076 | Yes |
| 44 | GTSE1 |  |  | 66 | 1.491 | 0.2112 | Yes |
| 45 | E2F8 |  |  | 67 | 1.488 | 0.2154 | Yes |
| 46 | CDK1 |  |  | 69 | 1.488 | 0.2194 | Yes |
| 47 | NRGN |  |  | 71 | 1.467 | 0.2234 | Yes |
| 48 | SMC2 |  |  | 73 | 1.466 | 0.2273 | Yes |
| 49 | SPC25 |  |  | 76 | 1.442 | 0.2310 | Yes |
| 50 | CHAF1A |  |  | 77 | 1.441 | 0.2351 | Yes |
| 51 | UBE2T |  |  | 79 | 1.432 | 0.2390 | Yes |
| 52 | FANCD2 |  |  | 81 | 1.423 | 0.2428 | Yes |
| 53 | FBXO5 |  |  | 82 | 1.422 | 0.2468 | Yes |
| 54 | KLHL23 |  |  | 83 | 1.421 | 0.2509 | Yes |
| 55 | CLSPN |  |  | 84 | 1.417 | 0.2549 | Yes |
| 56 | CIP2A |  |  | 86 | 1.410 | 0.2587 | Yes |
| 57 | MCM7 |  |  | 88 | 1.400 | 0.2624 | Yes |
| 58 | MCM8 |  |  | 89 | 1.397 | 0.2664 | Yes |
| 59 | CSRP2 |  |  | 90 | 1.396 | 0.2703 | Yes |
| 60 | MCM10 |  |  | 92 | 1.389 | 0.2741 | Yes |
| 61 | DEK |  |  | 94 | 1.384 | 0.2778 | Yes |
| 62 | CDCA2 |  |  | 96 | 1.382 | 0.2815 | Yes |
| 63 | RRM1 |  |  | 99 | 1.364 | 0.2850 | Yes |
| 64 | CARHSP1 |  |  | 101 | 1.362 | 0.2886 | Yes |
| 65 | OLFM1 |  |  | 102 | 1.361 | 0.2925 | Yes |
| 66 | POLD3 |  |  | 111 | 1.340 | 0.2947 | Yes |
| 67 | KIFC1 |  |  | 113 | 1.338 | 0.2983 | Yes |
| 68 | CENPM |  |  | 114 | 1.337 | 0.3020 | Yes |
| 69 | TACC3 |  |  | 116 | 1.334 | 0.3056 | Yes |
| 70 | CDCA5 |  |  | 123 | 1.326 | 0.3082 | Yes |
| 71 | SMC4 |  |  | 127 | 1.320 | 0.3113 | Yes |
| 72 | BARD1 |  |  | 132 | 1.310 | 0.3142 | Yes |
| 73 | MMS22L |  |  | 134 | 1.306 | 0.3177 | Yes |
| 74 | FEN1 |  |  | 139 | 1.296 | 0.3205 | Yes |
| 75 | RTKN2 |  |  | 143 | 1.285 | 0.3236 | Yes |
| 76 | LIG1 |  |  | 145 | 1.284 | 0.3270 | Yes |
| 77 | GMNN |  |  | 146 | 1.280 | 0.3306 | Yes |
| 78 | NCAPD3 |  |  | 155 | 1.261 | 0.3326 | Yes |
| 79 | LRRCC1 |  |  | 156 | 1.253 | 0.3361 | Yes |
| 80 | MIS18BP1 |  |  | 159 | 1.247 | 0.3393 | Yes |
| 81 | TMPO |  |  | 161 | 1.243 | 0.3426 | Yes |
| 82 | CENPK |  |  | 162 | 1.241 | 0.3461 | Yes |
| 83 | CDKN3 |  |  | 163 | 1.239 | 0.3496 | Yes |
| 84 | LMNB1 |  |  | 165 | 1.238 | 0.3529 | Yes |
| 85 | DNMT1 |  |  | 166 | 1.237 | 0.3564 | Yes |
| 86 | KIF23 |  |  | 167 | 1.235 | 0.3599 | Yes |
| 87 | NCAPH |  |  | 168 | 1.235 | 0.3634 | Yes |
| 88 | CDCA3 |  |  | 169 | 1.230 | 0.3669 | Yes |
| 89 | RFC4 |  |  | 171 | 1.227 | 0.3702 | Yes |
| 90 | NFIC |  |  | 172 | 1.227 | 0.3737 | Yes |
| 91 | GGH |  |  | 182 | 1.207 | 0.3752 | Yes |
| 92 | DBF4 |  |  | 189 | 1.197 | 0.3774 | Yes |
| 93 | KIF4A |  |  | 191 | 1.193 | 0.3806 | Yes |
| 94 | NUF2 |  |  | 192 | 1.189 | 0.3840 | Yes |
| 95 | CDT1 |  |  | 194 | 1.185 | 0.3871 | Yes |
| 96 | PXMP2 |  |  | 195 | 1.180 | 0.3905 | Yes |
| 97 | FANCI |  |  | 196 | 1.179 | 0.3938 | Yes |
| 98 | FANCA |  |  | 199 | 1.173 | 0.3967 | Yes |
| 99 | USP1 |  |  | 202 | 1.168 | 0.3996 | Yes |
| 100 | ZWINT |  |  | 204 | 1.166 | 0.4027 | Yes |
| 101 | RNASEH2B |  |  | 205 | 1.166 | 0.4060 | Yes |
| 102 | ASPM |  |  | 207 | 1.163 | 0.4091 | Yes |
| 103 | RBBP8 |  |  | 210 | 1.162 | 0.4120 | Yes |
| 104 | PRC1 |  |  | 211 | 1.161 | 0.4153 | Yes |
| 105 | KIF22 |  |  | 213 | 1.157 | 0.4184 | Yes |
| 106 | UBE2C |  |  | 214 | 1.154 | 0.4216 | Yes |
| 107 | PIGX |  |  | 219 | 1.150 | 0.4241 | Yes |
| 108 | NCAPG2 |  |  | 225 | 1.141 | 0.4263 | Yes |
| 109 | BTG3 |  |  | 226 | 1.141 | 0.4295 | Yes |
| 110 | CDC45 |  |  | 228 | 1.138 | 0.4326 | Yes |
| 111 | MCUB |  |  | 230 | 1.137 | 0.4356 | Yes |
| 112 | CDK2 |  |  | 233 | 1.134 | 0.4384 | Yes |
| 113 | ASF1B |  |  | 237 | 1.130 | 0.4410 | Yes |
| 114 | MCM2 |  |  | 238 | 1.130 | 0.4442 | Yes |
| 115 | RFC3 |  |  | 239 | 1.130 | 0.4474 | Yes |
| 116 | NDC80 |  |  | 240 | 1.129 | 0.4506 | Yes |
| 117 | MCM5 |  |  | 243 | 1.127 | 0.4534 | Yes |
| 118 | SGO2 |  |  | 244 | 1.126 | 0.4566 | Yes |
| 119 | TPX2 |  |  | 248 | 1.122 | 0.4591 | Yes |
| 120 | BRIP1 |  |  | 249 | 1.121 | 0.4623 | Yes |
| 121 | SMC1A |  |  | 251 | 1.120 | 0.4653 | Yes |
| 122 | SAC3D1 |  |  | 252 | 1.118 | 0.4685 | Yes |
| 123 | RAD21 |  |  | 256 | 1.109 | 0.4710 | Yes |
| 124 | TIMELESS |  |  | 259 | 1.105 | 0.4737 | Yes |
| 125 | NFATC2IP |  |  | 269 | 1.092 | 0.4750 | Yes |
| 126 | HAUS8 |  |  | 273 | 1.089 | 0.4774 | Yes |
| 127 | BRCA2 |  |  | 274 | 1.089 | 0.4805 | Yes |
| 128 | MCM6 |  |  | 282 | 1.080 | 0.4822 | Yes |
| 129 | CKAP2L |  |  | 285 | 1.078 | 0.4848 | Yes |
| 130 | HIRIP3 |  |  | 289 | 1.077 | 0.4873 | Yes |
| 131 | PAQR4 |  |  | 291 | 1.076 | 0.4901 | Yes |
| 132 | PRIM1 |  |  | 293 | 1.074 | 0.4929 | Yes |
| 133 | AURKB |  |  | 297 | 1.068 | 0.4954 | Yes |
| 134 | ARHGAP11A |  |  | 303 | 1.064 | 0.4974 | Yes |
| 135 | CCNA2 |  |  | 309 | 1.057 | 0.4993 | Yes |
| 136 | H1FX |  |  | 310 | 1.055 | 0.5023 | Yes |
| 137 | CKAP2 |  |  | 312 | 1.053 | 0.5051 | Yes |
| 138 | PSIP1 |  |  | 313 | 1.053 | 0.5081 | Yes |
| 139 | DUT |  |  | 314 | 1.050 | 0.5111 | Yes |
| 140 | GAMT |  |  | 327 | 1.040 | 0.5116 | Yes |
| 141 | CDC6 |  |  | 328 | 1.040 | 0.5145 | Yes |
| 142 | CDCA7 |  |  | 330 | 1.038 | 0.5173 | Yes |
| 143 | TMEM106C |  |  | 334 | 1.034 | 0.5196 | Yes |
| 144 | RFC2 |  |  | 337 | 1.025 | 0.5221 | Yes |
| 145 | PIMREG |  |  | 339 | 1.025 | 0.5248 | Yes |
| 146 | WDR76 |  |  | 350 | 1.015 | 0.5256 | Yes |
| 147 | EXO1 |  |  | 354 | 1.010 | 0.5279 | Yes |
| 148 | CENPX |  |  | 355 | 1.010 | 0.5307 | Yes |
| 149 | RBL1 |  |  | 358 | 0.999 | 0.5332 | Yes |
| 150 | GINS4 |  |  | 359 | 0.997 | 0.5360 | Yes |
| 151 | DNAJC9 |  |  | 373 | 0.979 | 0.5361 | Yes |
| 152 | PARP1 |  |  | 374 | 0.978 | 0.5389 | Yes |
| 153 | MYBL1 |  |  | 375 | 0.977 | 0.5417 | Yes |
| 154 | DSN1 |  |  | 377 | 0.975 | 0.5442 | Yes |
| 155 | PHF19 |  |  | 378 | 0.971 | 0.5470 | Yes |
| 156 | CASP8AP2 |  |  | 379 | 0.970 | 0.5497 | Yes |
| 157 | PSMC3IP |  |  | 382 | 0.968 | 0.5521 | Yes |
| 158 | GINS1 |  |  | 385 | 0.965 | 0.5544 | Yes |
| 159 | DEPDC1B |  |  | 387 | 0.963 | 0.5569 | Yes |
| 160 | UHRF1 |  |  | 390 | 0.958 | 0.5592 | Yes |
| 161 | ZNF367 |  |  | 394 | 0.956 | 0.5613 | Yes |
| 162 | RPA3 |  |  | 395 | 0.953 | 0.5640 | Yes |
| 163 | YEATS4 |  |  | 397 | 0.951 | 0.5665 | Yes |
| 164 | SLBP |  |  | 403 | 0.949 | 0.5682 | Yes |
| 165 | HJURP |  |  | 406 | 0.945 | 0.5705 | Yes |
| 166 | MASTL |  |  | 407 | 0.945 | 0.5731 | Yes |
| 167 | SPC24 |  |  | 414 | 0.938 | 0.5746 | Yes |
| 168 | NUDT1 |  |  | 417 | 0.936 | 0.5768 | Yes |
| 169 | MAD2L1 |  |  | 421 | 0.932 | 0.5788 | Yes |
| 170 | UBR7 |  |  | 423 | 0.932 | 0.5813 | Yes |
| 171 | DSCC1 |  |  | 427 | 0.926 | 0.5833 | Yes |
| 172 | KPNA2 |  |  | 435 | 0.919 | 0.5845 | Yes |
| 173 | RACGAP1 |  |  | 443 | 0.912 | 0.5856 | Yes |
| 174 | VRK1 |  |  | 445 | 0.911 | 0.5880 | Yes |
| 175 | CENPH |  |  | 448 | 0.908 | 0.5902 | Yes |
| 176 | ORC6 |  |  | 449 | 0.908 | 0.5928 | Yes |
| 177 | METRN |  |  | 450 | 0.907 | 0.5953 | Yes |
| 178 | ECI1 |  |  | 462 | 0.901 | 0.5956 | Yes |
| 179 | SHCBP1 |  |  | 465 | 0.900 | 0.5978 | Yes |
| 180 | POC1A |  |  | 467 | 0.896 | 0.6001 | Yes |
| 181 | LSM3 |  |  | 468 | 0.892 | 0.6026 | Yes |
| 182 | MGME1 |  |  | 469 | 0.892 | 0.6052 | Yes |
| 183 | CENPJ |  |  | 479 | 0.885 | 0.6058 | Yes |
| 184 | MND1 |  |  | 480 | 0.885 | 0.6084 | Yes |
| 185 | ABHD3 |  |  | 494 | 0.872 | 0.6082 | Yes |
| 186 | H2AFY |  |  | 497 | 0.868 | 0.6102 | Yes |
| 187 | PAXX |  |  | 501 | 0.864 | 0.6121 | Yes |
| 188 | PKMYT1 |  |  | 506 | 0.859 | 0.6137 | Yes |
| 189 | DCK |  |  | 516 | 0.854 | 0.6143 | Yes |
| 190 | RFC5 |  |  | 527 | 0.849 | 0.6146 | Yes |
| 191 | LMNB2 |  |  | 534 | 0.846 | 0.6158 | Yes |
| 192 | BUB1B |  |  | 535 | 0.846 | 0.6182 | Yes |
| 193 | BIRC5 |  |  | 541 | 0.842 | 0.6196 | Yes |
| 194 | TCF19 |  |  | 542 | 0.842 | 0.6220 | Yes |
| 195 | CEP152 |  |  | 547 | 0.834 | 0.6235 | Yes |
| 196 | SLF1 |  |  | 549 | 0.831 | 0.6257 | Yes |
| 197 | PRKDC |  |  | 554 | 0.828 | 0.6272 | Yes |
| 198 | CENPQ |  |  | 557 | 0.825 | 0.6291 | Yes |
| 199 | TEDC1 |  |  | 559 | 0.825 | 0.6313 | Yes |
| 200 | WDHD1 |  |  | 562 | 0.823 | 0.6332 | Yes |
| 201 | CMC2 |  |  | 580 | 0.812 | 0.6320 | Yes |
| 202 | DIAPH3 |  |  | 585 | 0.808 | 0.6335 | Yes |
| 203 | TK1 |  |  | 591 | 0.806 | 0.6348 | Yes |
| 204 | MSH6 |  |  | 593 | 0.804 | 0.6369 | Yes |
| 205 | RAD18 |  |  | 603 | 0.799 | 0.6373 | Yes |
| 206 | ACYP1 |  |  | 609 | 0.794 | 0.6385 | Yes |
| 207 | RBBP4 |  |  | 610 | 0.794 | 0.6408 | Yes |
| 208 | CDCA4 |  |  | 615 | 0.790 | 0.6422 | Yes |
| 209 | OIP5 |  |  | 620 | 0.788 | 0.6436 | Yes |
| 210 | TMEM107 |  |  | 633 | 0.782 | 0.6434 | Yes |
| 211 | SIVA1 |  |  | 646 | 0.774 | 0.6431 | Yes |
| 212 | HNRNPD |  |  | 659 | 0.769 | 0.6429 | Yes |
| 213 | C3orf14 |  |  | 662 | 0.769 | 0.6446 | Yes |
| 214 | SKA3 |  |  | 668 | 0.765 | 0.6458 | Yes |
| 215 | WDR34 |  |  | 671 | 0.762 | 0.6475 | Yes |
| 216 | RECQL4 |  |  | 676 | 0.761 | 0.6489 | Yes |
| 217 | ARL6IP6 |  |  | 702 | 0.745 | 0.6459 | Yes |
| 218 | UNG |  |  | 703 | 0.745 | 0.6480 | Yes |
| 219 | SMC3 |  |  | 708 | 0.743 | 0.6493 | Yes |
| 220 | TFDP1 |  |  | 711 | 0.742 | 0.6510 | Yes |
| 221 | ATAD5 |  |  | 721 | 0.739 | 0.6513 | Yes |
| 222 | ANP32E |  |  | 722 | 0.739 | 0.6534 | Yes |
| 223 | KDELC2 |  |  | 723 | 0.738 | 0.6554 | Yes |
| 224 | MAD2L2 |  |  | 724 | 0.736 | 0.6575 | Yes |
| 225 | TEX30 |  |  | 730 | 0.730 | 0.6586 | Yes |
| 226 | HAT1 |  |  | 741 | 0.727 | 0.6586 | Yes |
| 227 | PIN1 |  |  | 746 | 0.725 | 0.6598 | Yes |
| 228 | NABP2 |  |  | 769 | 0.714 | 0.6574 | Yes |
| 229 | INAFM1 |  |  | 774 | 0.713 | 0.6586 | Yes |
| 230 | TUBG1 |  |  | 776 | 0.712 | 0.6604 | Yes |
| 231 | CHEK1 |  |  | 779 | 0.709 | 0.6620 | Yes |
| 232 | ACTL6A |  |  | 780 | 0.709 | 0.6640 | Yes |
| 233 | CCDC15 |  |  | 782 | 0.709 | 0.6658 | Yes |
| 234 | CBX5 |  |  | 783 | 0.708 | 0.6678 | Yes |
| 235 | RAD54L |  |  | 790 | 0.704 | 0.6686 | Yes |
| 236 | SNRNP25 |  |  | 795 | 0.702 | 0.6698 | Yes |
| 237 | HPF1 |  |  | 796 | 0.702 | 0.6718 | Yes |
| 238 | DTYMK |  |  | 800 | 0.700 | 0.6731 | Yes |
| 239 | TRIP13 |  |  | 804 | 0.698 | 0.6745 | Yes |
| 240 | MMD |  |  | 806 | 0.697 | 0.6763 | Yes |
| 241 | NME4 |  |  | 819 | 0.692 | 0.6758 | Yes |
| 242 | RBBP7 |  |  | 829 | 0.687 | 0.6759 | Yes |
| 243 | LRRC45 |  |  | 831 | 0.687 | 0.6777 | Yes |
| 244 | TUBB |  |  | 836 | 0.685 | 0.6788 | Yes |
| 245 | SUZ12 |  |  | 846 | 0.680 | 0.6789 | Yes |
| 246 | ALYREF |  |  | 847 | 0.680 | 0.6808 | Yes |
| 247 | H2AFZ |  |  | 851 | 0.677 | 0.6821 | Yes |
| 248 | RDX |  |  | 858 | 0.675 | 0.6828 | Yes |
| 249 | PARP2 |  |  | 864 | 0.673 | 0.6837 | Yes |
| 250 | SKA1 |  |  | 868 | 0.672 | 0.6850 | Yes |
| 251 | DDB2 |  |  | 872 | 0.669 | 0.6863 | Yes |
| 252 | C19orf48 |  |  | 893 | 0.661 | 0.6841 | Yes |
| 253 | BCL7C |  |  | 898 | 0.660 | 0.6851 | Yes |
| 254 | FAM161A |  |  | 906 | 0.658 | 0.6856 | Yes |
| 255 | MELK |  |  | 915 | 0.653 | 0.6858 | Yes |
| 256 | H2AFV |  |  | 933 | 0.647 | 0.6842 | Yes |
| 257 | TMEM237 |  |  | 952 | 0.639 | 0.6823 | Yes |
| 258 | CENPN |  |  | 953 | 0.638 | 0.6841 | Yes |
| 259 | HNRNPA3 |  |  | 954 | 0.638 | 0.6859 | Yes |
| 260 | TMEM97 |  |  | 963 | 0.636 | 0.6861 | Yes |
| 261 | PEG10 |  |  | 964 | 0.636 | 0.6879 | Yes |
| 262 | CKS2 |  |  | 966 | 0.634 | 0.6895 | Yes |
| 263 | PRIM2 |  |  | 968 | 0.633 | 0.6911 | Yes |
| 264 | HAUS1 |  |  | 987 | 0.624 | 0.6892 | Yes |
| 265 | LRR1 |  |  | 991 | 0.623 | 0.6903 | Yes |
| 266 | ARRB2 |  |  | 994 | 0.622 | 0.6917 | Yes |
| 267 | RANBP1 |  |  | 999 | 0.620 | 0.6926 | Yes |
| 268 | LSM4 |  |  | 1003 | 0.619 | 0.6938 | Yes |
| 269 | TCTEX1D2 |  |  | 1008 | 0.618 | 0.6947 | Yes |
| 270 | MIS18A |  |  | 1023 | 0.614 | 0.6936 | Yes |
| 271 | NRM |  |  | 1031 | 0.613 | 0.6939 | Yes |
| 272 | MNS1 |  |  | 1034 | 0.613 | 0.6952 | Yes |
| 273 | PAFAH1B3 |  |  | 1039 | 0.612 | 0.6962 | Yes |
| 274 | ZWILCH |  |  | 1048 | 0.609 | 0.6963 | Yes |
| 275 | CEP57 |  |  | 1053 | 0.607 | 0.6972 | Yes |
| 276 | CDC7 |  |  | 1060 | 0.604 | 0.6977 | Yes |
| 277 | BAZ1B |  |  | 1072 | 0.601 | 0.6971 | Yes |
| 278 | DONSON |  |  | 1082 | 0.597 | 0.6970 | Yes |
| 279 | PIDD1 |  |  | 1092 | 0.594 | 0.6968 | Yes |
| 280 | MSH2 |  |  | 1107 | 0.590 | 0.6956 | Yes |
| 281 | SRR |  |  | 1130 | 0.585 | 0.6928 | Yes |
| 282 | TEDC2 |  |  | 1142 | 0.579 | 0.6922 | Yes |
| 283 | HMGB3 |  |  | 1154 | 0.577 | 0.6916 | Yes |
| 284 | POLA2 |  |  | 1161 | 0.574 | 0.6920 | Yes |
| 285 | DEPDC1 |  |  | 1179 | 0.569 | 0.6902 | Yes |
| 286 | ITGB3BP |  |  | 1180 | 0.568 | 0.6918 | Yes |
| 287 | ANP32B |  |  | 1187 | 0.566 | 0.6922 | Yes |
| 288 | MEA1 |  |  | 1197 | 0.562 | 0.6919 | Yes |
| 289 | PARPBP |  |  | 1210 | 0.560 | 0.6911 | Yes |
| 290 | TMEM54 |  |  | 1215 | 0.559 | 0.6918 | Yes |
| 291 | HNRNPF |  |  | 1216 | 0.559 | 0.6934 | Yes |
| 292 | CBR3 |  |  | 1221 | 0.556 | 0.6942 | Yes |
| 293 | MTFR2 |  |  | 1222 | 0.555 | 0.6957 | Yes |
| 294 | EXOSC8 |  |  | 1230 | 0.553 | 0.6959 | Yes |
| 295 | RAD1 |  |  | 1234 | 0.552 | 0.6968 | Yes |
| 296 | SPATA33 |  |  | 1251 | 0.546 | 0.6951 | Yes |
| 297 | PRTFDC1 |  |  | 1254 | 0.545 | 0.6963 | Yes |
| 298 | CSE1L |  |  | 1259 | 0.543 | 0.6970 | Yes |
| 299 | RMI2 |  |  | 1261 | 0.542 | 0.6983 | Yes |
| 300 | CEP135 |  |  | 1264 | 0.541 | 0.6995 | Yes |
| 301 | CCDC167 |  |  | 1303 | 0.531 | 0.6932 | No |
| 302 | RPA1 |  |  | 1307 | 0.530 | 0.6941 | No |
| 303 | KIF2C |  |  | 1309 | 0.530 | 0.6954 | No |
| 304 | RAC3 |  |  | 1339 | 0.523 | 0.6910 | No |
| 305 | CMSS1 |  |  | 1350 | 0.520 | 0.6904 | No |
| 306 | GGCT |  |  | 1414 | 0.501 | 0.6790 | No |
| 307 | CNPY3 |  |  | 1453 | 0.492 | 0.6726 | No |
| 308 | ACADM |  |  | 1475 | 0.487 | 0.6698 | No |
| 309 | CKLF |  |  | 1480 | 0.486 | 0.6703 | No |
| 310 | PGRMC1 |  |  | 1498 | 0.480 | 0.6682 | No |
| 311 | SUPT16H |  |  | 1500 | 0.480 | 0.6694 | No |
| 312 | CKS1B |  |  | 1502 | 0.479 | 0.6705 | No |
| 313 | CHTF18 |  |  | 1507 | 0.479 | 0.6711 | No |
| 314 | C5orf34 |  |  | 1514 | 0.478 | 0.6712 | No |
| 315 | LSM8 |  |  | 1524 | 0.476 | 0.6707 | No |
| 316 | NUP85 |  |  | 1529 | 0.475 | 0.6712 | No |
| 317 | ENOPH1 |  |  | 1539 | 0.471 | 0.6707 | No |
| 318 | HNRNPAB |  |  | 1541 | 0.471 | 0.6719 | No |
| 319 | POP7 |  |  | 1591 | 0.461 | 0.6632 | No |
| 320 | UBA2 |  |  | 1612 | 0.457 | 0.6604 | No |
| 321 | SSRP1 |  |  | 1634 | 0.451 | 0.6574 | No |
| 322 | TUBB4B |  |  | 1659 | 0.444 | 0.6538 | No |
| 323 | NR2C2AP |  |  | 1690 | 0.438 | 0.6489 | No |
| 324 | DERA |  |  | 1749 | 0.428 | 0.6383 | No |
| 325 | FANCG |  |  | 1763 | 0.425 | 0.6369 | No |
| 326 | DCTPP1 |  |  | 1796 | 0.418 | 0.6315 | No |
| 327 | SNX5 |  |  | 1849 | 0.410 | 0.6221 | No |
| 328 | NCAPH2 |  |  | 1915 | 0.401 | 0.6100 | No |
| 329 | CENPW |  |  | 1959 | 0.395 | 0.6024 | No |
| 330 | CCND3 |  |  | 1991 | 0.390 | 0.5972 | No |
| 331 | TMX1 |  |  | 2012 | 0.386 | 0.5942 | No |
| 332 | PA2G4 |  |  | 2112 | 0.372 | 0.5751 | No |
| 333 | REXO5 |  |  | 2280 | 0.353 | 0.5420 | No |
| 334 | POLR2I |  |  | 2316 | 0.347 | 0.5359 | No |
| 335 | NFYB |  |  | 2330 | 0.344 | 0.5342 | No |
| 336 | MACROD1 |  |  | 2336 | 0.344 | 0.5342 | No |
| 337 | COA1 |  |  | 2406 | 0.334 | 0.5210 | No |
| 338 | SCARA3 |  |  | 2409 | 0.334 | 0.5216 | No |
| 339 | CHCHD3 |  |  | 2434 | 0.330 | 0.5176 | No |
| 340 | PPIF |  |  | 2478 | 0.325 | 0.5098 | No |
| 341 | RPA2 |  |  | 2570 | 0.314 | 0.4921 | No |
| 342 | IL17RB |  |  | 2596 | 0.311 | 0.4879 | No |
| 343 | AHCY |  |  | 2598 | 0.311 | 0.4886 | No |
| 344 | AKR7A2 |  |  | 2611 | 0.309 | 0.4870 | No |
| 345 | MRM2 |  |  | 2622 | 0.307 | 0.4859 | No |
| 346 | CSNK2B |  |  | 2627 | 0.307 | 0.4859 | No |
| 347 | BUB3 |  |  | 2806 | 0.288 | 0.4505 | No |
| 348 | PRADC1 |  |  | 2845 | 0.285 | 0.4435 | No |
| 349 | SAE1 |  |  | 2872 | 0.283 | 0.4390 | No |
| 350 | MTHFD1 |  |  | 2917 | 0.277 | 0.4309 | No |
| 351 | FKBP3 |  |  | 2946 | 0.274 | 0.4259 | No |
| 352 | AUNIP |  |  | 2977 | 0.272 | 0.4206 | No |
| 353 | CALU |  |  | 3001 | 0.269 | 0.4167 | No |
| 354 | CHAF1B |  |  | 3047 | 0.264 | 0.4083 | No |
| 355 | TMUB1 |  |  | 3051 | 0.264 | 0.4084 | No |
| 356 | DPM2 |  |  | 3082 | 0.261 | 0.4030 | No |
| 357 | VOPP1 |  |  | 3125 | 0.258 | 0.3952 | No |
| 358 | HSPB11 |  |  | 3163 | 0.254 | 0.3884 | No |
| 359 | RALY |  |  | 3167 | 0.254 | 0.3885 | No |
| 360 | TECR |  |  | 3183 | 0.252 | 0.3861 | No |
| 361 | HAUS2 |  |  | 3186 | 0.251 | 0.3864 | No |
| 362 | PGK1 |  |  | 3286 | -0.263 | 0.3670 | No |
| 363 | ITGB1BP1 |  |  | 3341 | -0.274 | 0.3568 | No |
| 364 | NUDC |  |  | 3349 | -0.275 | 0.3561 | No |
| 365 | JPT2 |  |  | 3459 | -0.292 | 0.3348 | No |
| 366 | CCT2 |  |  | 3510 | -0.301 | 0.3254 | No |
| 367 | EEF1B2 |  |  | 3605 | -0.316 | 0.3072 | No |
| 368 | NME1 |  |  | 3615 | -0.317 | 0.3062 | No |
| 369 | ATP5MC1 |  |  | 3899 | -0.367 | 0.2496 | No |
| 370 | TUBB6 |  |  | 3904 | -0.368 | 0.2498 | No |
| 371 | SUMO3 |  |  | 3910 | -0.369 | 0.2499 | No |
| 372 | TUBA1C |  |  | 3957 | -0.379 | 0.2416 | No |
| 373 | CCT5 |  |  | 3958 | -0.379 | 0.2426 | No |
| 374 | SNRPF |  |  | 4037 | -0.397 | 0.2279 | No |
| 375 | SHMT2 |  |  | 4131 | -0.417 | 0.2101 | No |
| 376 | PLP2 |  |  | 4366 | -0.490 | 0.1638 | No |
| 377 | CCT4 |  |  | 4407 | -0.503 | 0.1571 | No |
| 378 | TMEM14C |  |  | 4413 | -0.505 | 0.1575 | No |
| 379 | PSMB9 |  |  | 4503 | -0.543 | 0.1409 | No |
| 380 | PSMG1 |  |  | 4573 | -0.574 | 0.1285 | No |
| 381 | PSMA3 |  |  | 4650 | -0.618 | 0.1148 | No |
| 382 | KDELR2 |  |  | 4700 | -0.645 | 0.1066 | No |
| 383 | PFKP |  |  | 4734 | -0.666 | 0.1018 | No |
| 384 | ARPC1B |  |  | 4818 | -0.730 | 0.0869 | No |
| 385 | SMS |  |  | 4842 | -0.750 | 0.0844 | No |
| 386 | CLIC1 |  |  | 4899 | -0.809 | 0.0752 | No |
| 387 | HMGA1 |  |  | 4962 | -0.878 | 0.0651 | No |
| 388 | FUCA2 |  |  | 4977 | -0.896 | 0.0648 | No |
Table: GSEA details [plain text format]

  

Fig 2: DOROSZKO\_U3180MG\_CLUSTER\_3: Random ES distribution      
 Gene set null distribution of ES for **DOROSZKO\_U3180MG\_CLUSTER\_3**
